# 4-Methyl-2,6-bis­(pyrrolidin-1-yl)pyrimidine

**DOI:** 10.1107/S160053681204740X

**Published:** 2012-11-24

**Authors:** S. Sreenivasa, K. E. ManojKumar, M. Shet Prakash, P. A. Suchetan, B. S. Palakshamurthy

**Affiliations:** a Department of Studies and Research in Chemistry, Tumkur University, Tumkur, Karnataka 572 103, India; b Department of Studies and Research in Chemistry, U.C.S., Tumkur University, Tumkur, Karnataka 572 103, India; c Department of Studies and Research in Physics, U.C.S., Tumkur University, Tumkur, Karnataka 572 103, India

## Abstract

In the crystal of the title compound, C_13_H_20_N_4_, the mol­ecule is nearly planar, the dihedral angles between the pyrimidine and the two pyrrolidine rings being 4.71 (2) and 4.50 (2)°. The crystal features inversion-related dimers linked by pairs of C—H⋯N hydrogen bonds generating *R*_2_^2^(16) patterns. The dimeric units are further linked into *C*(6) chains *via* an additional C—H⋯N hydrogen bond.

## Related literature

For the synthesis and biological activity of pyrrolidine derivatives, see: Li *et al.* (2006 ▶); Lokhande *et al.* (2003 ▶); Imamura *et al.* (2004 ▶); Wyrzykiewicz, *et al.* (1993 ▶) and of pyrimidine derivatives, see: Holla *et al.* (2006 ▶); Zhao *et al.* (2007 ▶); Sondhi *et al.* (2005 ▶); Khalifa *et al.* (2005 ▶). For the graph-set description of hydrogen-bond motifs, see: Etter (1990 ▶); Bernstein *et al.* (1995 ▶).
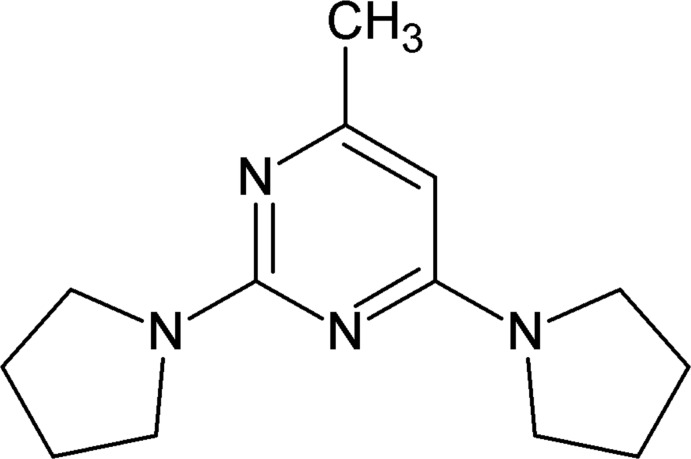


## Experimental

### 

#### Crystal data

C_13_H_20_N_4_
*M* *_r_* = 232.33
Triclinic, 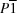

*a* = 6.344 (3) Å
*b* = 8.766 (4) Å
*c* = 12.056 (6) Å
α = 79.10 (3)°
β = 86.05 (3)°
γ = 85.72 (3)°
*V* = 655.6 (6) Å^3^
*Z* = 2
Mo *K*α radiation
μ = 0.07 mm^−1^
*T* = 296 K
0.2 × 0.18 × 0.02 mm

#### Data collection

Bruker SMART X2S diffractometer
Absorption correction: multi-scan (*SADABS*; Bruker, 2009 ▶) *T*_min_ = 0.986, *T*_max_ = 0.999
8712 measured reflections
2302 independent reflections
1600 reflections with *I* > 2σ(*I*)
*R*_int_ = 0.022

#### Refinement

*R*[*F* ^2^ > 2σ(*F* ^2^)] = 0.066
*wR*(*F* ^2^) = 0.227
*S* = 1.13
2302 reflections
155 parameters
H-atom parameters constrained
Δρ_max_ = 0.24 e Å^−3^
Δρ_min_ = −0.20 e Å^−3^

### 

Data collection: *APEX2* (Bruker, 2009 ▶); cell refinement: *APEX2* and *SAINT* (Bruker, 2009 ▶); data reduction: *SAINT*; program(s) used to solve structure: *SHELXS97* (Sheldrick, 2008 ▶); program(s) used to refine structure: *SHELXL97* (Sheldrick, 2008 ▶); molecular graphics: *ORTEP-3* (Farrugia, 2012 ▶); software used to prepare material for publication: *SHELXL97*.

## Supplementary Material

Crystal structure: contains datablock(s) I, global. DOI: 10.1107/S160053681204740X/bv2214sup1.cif

Structure factors: contains datablock(s) I. DOI: 10.1107/S160053681204740X/bv2214Isup2.hkl

Supplementary material file. DOI: 10.1107/S160053681204740X/bv2214Isup3.cml

Additional supplementary materials:  crystallographic information; 3D view; checkCIF report

## Figures and Tables

**Table 1:** Hydrogen-bond geometry (Å, °)

*D*—H⋯*A*	*D*—H	H⋯*A*	*D*⋯*A*	*D*—H⋯*A*
C2—H2*B*⋯N3^i^	0.97 (1)	2.82	3.793 (2)	175
C9—H9*C*⋯N2^ii^	0.96 (1)	2.93	3.742 (2)	143

Symmetry codes: (i) 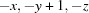; (ii) 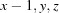.

**Fig. 1.**
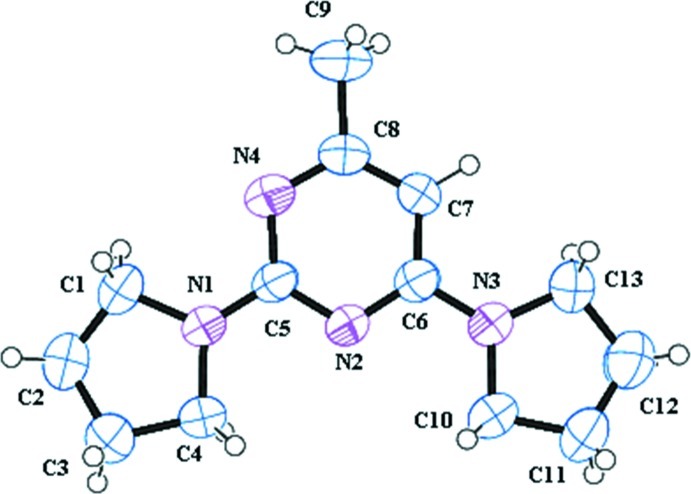
Molecular structure of the title compound, showing the atom-labeling scheme. Displacement ellipsoids are drawn at the 50% probability level.

**Fig. 2.**
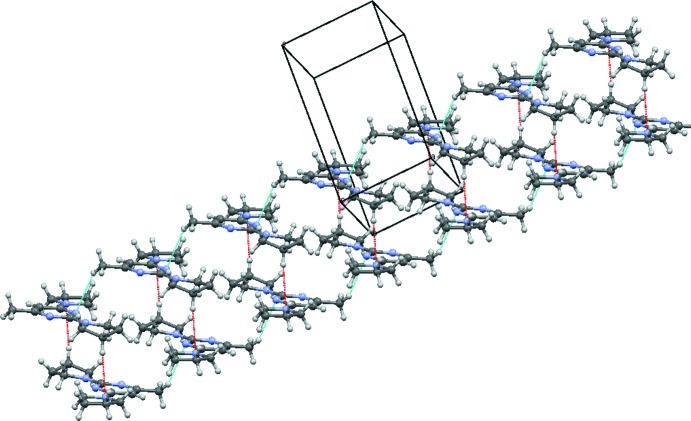
Molecular packing in the title compound. Hydrogen bonds are shown as dashed lines.

## supplementary crystallographic information

### Comment

Organic compounds with the pyrrolidine ring system are known to display a wide range of biological activities such as antitumor (Li *et al.*, 2006), antimicrobial (Lokhande *et al.*, 2003), anti- HIV-1 (Imamura *et al.*, 2004). Similarly pyrimidine derivatives exhibit a range of pharmacological activities such as antibacterial (Wyrzykiewicz *et al.*, 1993), antifungal (Holla *et al.*, 2006), anticancer (Zhao *et al.*, 2007), anti inflammatory (Sondhi *et al.*, 2005) and cardioprotective effects (Khalifa *et al.*, 2005). In this view the title compound was synthesized to study its crystal structure.

The compound crystallizes in triclinic P-1 space group. In the crystal structure, the molecule is nearly planar, with the dihedral angles between the pyrimidine and the two pyrrolidine rings being 4.71 (2) and 4.50 (2)°. Further, the dihedral angle between the two pyrrolidine rings is 18.70 (2)°. The crystal structure features inversion-related dimers linked by pairs of C—H···N hydrogen bonds generating *R*_2_^2^(16) patterns (Etter, 1990; Bernstein *et al.*, 1995). The dimeric units are further linked into C(6) chains *via* an additional C—H···N hydrogen bond.

### Experimental

2,4-Dichloro-6-methyl pyrimidine (3.11 mmol), pyrrolidine (6.83 mmol), and triethylamine (12.4 mmol) and 5 ml acetonitrile were taken in a microwave seal tube. The reaction mixture was irradiated with microwave for 90 min. The reaction was monitored by TLC with 30% ethyl acetate in petroleum ether. The solvent was removed and the residue dissolved in dichloromethane, purified by column chromatography, and the collected fraction was concentrated under reduced pressure. Single crystals employed in X-ray diffraction studies were obtained from slow evaporation of the solvent from the solution of the compound in ethyl acetate-petroleum ether at room temperature.

### Refinement

The H atoms were positioned with idealized geometry using a riding model with C—H = 0.93 - 0.97 Å. The isotropic displacement parameters for all H atoms were set to 1.2 times of the *U*_eq_ of the parent atom (1.5 times of the *U*_eq_ of the parent atom for CH_3_).

### Crystal data

**Table:**

C_13_H_20_N_4_	*F*(000) = 252
*M**_r_* = 232.33	Colourless
Triclinic, *P*1	*D*_x_ = 1.177 Mg m^−^^3^
Hall symbol: -P 1	Melting point: 446 K
*a* = 6.344 (3) Å	Mo *K*α radiation, λ = 0.71073 Å
*b* = 8.766 (4) Å	Cell parameters from 1600 reflections
*c* = 12.056 (6) Å	θ = 25°
α = 79.10 (3)°	µ = 0.07 mm^−^^1^
β = 86.05 (3)°	*T* = 296 K
γ = 85.72 (3)°	Prism, colorless
*V* = 655.6 (6) Å^3^	0.2 × 0.18 × 0.02 mm
*Z* = 2	

### Data collection

**Table:**

Bruker SMART X2S diffractometer	2302 independent reflections
Radiation source: fine-focus steel tube	1600 reflections with *I* > 2σ(*I*)
Graphite monochromator	*R*_int_ = 0.022
Detector resolution: 1.03 pixels mm^-1^	θ_max_ = 25°, θ_min_ = 1.7°
phi and ω scans	*h* = −7→7
Absorption correction: multi-scan (*SADABS*; Bruker, 2009)	*k* = −10→10
*T*_min_ = 0.986, *T*_max_ = 0.999	*l* = −14→14
8712 measured reflections	

### Refinement

**Table:**

Refinement on *F*^2^	Primary atom site location: structure-invariant direct methods
Least-squares matrix: full	Secondary atom site location: difference Fourier map
*R*[*F*^2^ > 2σ(*F*^2^)] = 0.066	Hydrogen site location: inferred from neighbouring sites
*wR*(*F*^2^) = 0.227	H-atom parameters constrained
*S* = 1.13	*w* = 1/[σ^2^(*F*_o_^2^) + (0.1307*P*)^2^ + 0.0781*P*] where *P* = (*F*_o_^2^ + 2*F*_c_^2^)/3
2302 reflections	(Δ/σ)_max_ = 0.009
155 parameters	Δρ_max_ = 0.24 e Å^−^^3^
0 restraints	Δρ_min_ = −0.20 e Å^−^^3^
0 constraints	

### Special details

**Table:**

Geometry. All e.s.d.'s (except the e.s.d. in the dihedral angle between two l.s. planes) are estimated using the full covariance matrix. The cell e.s.d.'s are taken into account individually in the estimation of e.s.d.'s in distances, angles and torsion angles; correlations between e.s.d.'s in cell parameters are only used when they are defined by crystal symmetry. An approximate (isotropic) treatment of cell e.s.d.'s is used for estimating e.s.d.'s involving l.s. planes.
Refinement. Refinement of *F*^2^ against ALL reflections. The weighted *R*-factor *wR* and goodness of fit *S* are based on *F*^2^, conventional *R*-factors *R* are based on *F*, with *F* set to zero for negative *F*^2^. The threshold expression of *F*^2^ > σ(*F*^2^) is used only for calculating *R*-factors(gt) *etc*. and is not relevant to the choice of reflections for refinement. *R*-factors based on *F*^2^ are statistically about twice as large as those based on *F*, and *R*- factors based on ALL data will be even larger.

### Fractional atomic coordinates and isotropic or equivalent isotropic displacement parameters (Å^2^)

**Table:**

	*x*	*y*	*z*	*U*_iso_*/*U*_eq_	
N1	0.1352 (3)	0.2849 (2)	0.09361 (17)	0.0628 (6)	
N2	0.0463 (3)	0.4541 (2)	0.21449 (16)	0.0540 (5)	
N3	−0.0420 (3)	0.6226 (2)	0.33585 (17)	0.0645 (6)	
N4	−0.1692 (3)	0.2415 (2)	0.21113 (17)	0.0642 (6)	
C3	0.4251 (5)	0.2730 (3)	−0.0331 (3)	0.0869 (9)	
H3A	0.4825	0.3359	−0.102	0.104*	
H3B	0.5404	0.2102	0.0052	0.104*	
C4	0.3118 (4)	0.3748 (3)	0.0424 (2)	0.0619 (7)	
H4A	0.4029	0.3928	0.0994	0.074*	
H4B	0.2625	0.4743	−0.001	0.074*	
C5	0.0014 (3)	0.3283 (2)	0.17442 (19)	0.0535 (6)	
C6	−0.0892 (3)	0.4968 (2)	0.29357 (18)	0.0534 (6)	
C13	−0.1759 (4)	0.6913 (3)	0.4170 (2)	0.0730 (7)	
H13A	−0.1876	0.6202	0.489	0.088*	
H13B	−0.3164	0.72	0.3899	0.088*	
C12	−0.0658 (6)	0.8320 (4)	0.4279 (3)	0.1077 (12)	
H12A	−0.1492	0.9254	0.3963	0.129*	
H12B	−0.0487	0.8332	0.507	0.129*	
C2	0.2664 (5)	0.1735 (3)	−0.0592 (3)	0.0875 (9)	
H2A	0.3319	0.0727	−0.0683	0.105*	
H2B	0.2005	0.2212	−0.1287	0.105*	
C1	0.1062 (5)	0.1559 (3)	0.0377 (2)	0.0761 (8)	
H1A	−0.0358	0.162	0.0114	0.091*	
H1B	0.1307	0.0571	0.0885	0.091*	
C10	0.1473 (4)	0.7058 (3)	0.2981 (2)	0.0695 (7)	
H10A	0.1459	0.7517	0.2183	0.083*	
H10B	0.2742	0.6376	0.3112	0.083*	
C9	−0.4898 (4)	0.2017 (3)	0.3329 (2)	0.0803 (8)	
H9A	−0.4633	0.1377	0.4051	0.121*	
H9B	−0.5157	0.1369	0.28	0.121*	
H9C	−0.6114	0.2717	0.3406	0.121*	
C7	−0.2678 (3)	0.4204 (3)	0.33417 (18)	0.0538 (6)	
H7	−0.361	0.4543	0.3887	0.065*	
C8	−0.3015 (3)	0.2932 (3)	0.29089 (19)	0.0567 (6)	
C11	0.1343 (7)	0.8278 (5)	0.3691 (3)	0.1257 (14)	
H11A	0.2446	0.8058	0.4228	0.151*	
H11B	0.156	0.9284	0.3216	0.151*	

### Atomic displacement parameters (Å^2^)

**Table:**

	*U*^11^	*U*^22^	*U*^33^	*U*^12^	*U*^13^	*U*^23^
N1	0.0602 (12)	0.0608 (11)	0.0737 (13)	−0.0221 (9)	0.0104 (10)	−0.0268 (10)
N2	0.0506 (10)	0.0520 (10)	0.0626 (11)	−0.0116 (8)	−0.0004 (8)	−0.0165 (8)
N3	0.0629 (12)	0.0625 (11)	0.0740 (13)	−0.0178 (9)	0.0070 (10)	−0.0262 (10)
N4	0.0583 (12)	0.0643 (12)	0.0729 (13)	−0.0192 (9)	−0.0014 (10)	−0.0147 (10)
C3	0.091 (2)	0.0830 (18)	0.0925 (19)	−0.0260 (15)	0.0268 (16)	−0.0346 (15)
C4	0.0580 (14)	0.0593 (13)	0.0705 (15)	−0.0163 (11)	0.0054 (11)	−0.0156 (11)
C5	0.0503 (12)	0.0501 (12)	0.0620 (13)	−0.0119 (9)	−0.0048 (10)	−0.0114 (10)
C6	0.0514 (12)	0.0509 (12)	0.0585 (13)	−0.0055 (9)	−0.0055 (10)	−0.0101 (10)
C13	0.0802 (17)	0.0687 (15)	0.0744 (16)	−0.0084 (13)	0.0074 (13)	−0.0268 (13)
C12	0.101 (2)	0.089 (2)	0.148 (3)	−0.0174 (17)	0.020 (2)	−0.064 (2)
C2	0.094 (2)	0.0767 (18)	0.101 (2)	−0.0113 (15)	0.0081 (17)	−0.0419 (16)
C1	0.0868 (18)	0.0655 (15)	0.0851 (17)	−0.0225 (13)	0.0056 (15)	−0.0336 (13)
C10	0.0694 (15)	0.0633 (14)	0.0805 (17)	−0.0199 (12)	−0.0011 (13)	−0.0202 (13)
C9	0.0626 (15)	0.0877 (18)	0.0879 (18)	−0.0281 (13)	0.0078 (13)	−0.0044 (15)
C7	0.0492 (12)	0.0566 (12)	0.0560 (13)	−0.0066 (10)	0.0063 (10)	−0.0135 (10)
C8	0.0467 (12)	0.0611 (13)	0.0599 (13)	−0.0095 (10)	−0.0023 (10)	−0.0026 (11)
C11	0.152 (3)	0.120 (3)	0.129 (3)	−0.076 (2)	0.049 (3)	−0.077 (2)

### Geometric parameters (Å, º)

**Table:**

N1—C5	1.339 (3)	C12—C11	1.412 (5)
N1—C1	1.451 (3)	C12—H12A	0.97
N1—C4	1.452 (3)	C12—H12B	0.97
N2—C6	1.328 (3)	C2—C1	1.488 (4)
N2—C5	1.341 (3)	C2—H2A	0.97
N3—C6	1.360 (3)	C2—H2B	0.97
N3—C13	1.440 (3)	C1—H1A	0.97
N3—C10	1.453 (3)	C1—H1B	0.97
N4—C8	1.353 (3)	C10—C11	1.486 (4)
N4—C5	1.369 (3)	C10—H10A	0.97
C3—C2	1.467 (4)	C10—H10B	0.97
C3—C4	1.505 (3)	C9—C8	1.494 (3)
C3—H3A	0.97	C9—H9A	0.96
C3—H3B	0.97	C9—H9B	0.96
C4—H4A	0.97	C9—H9C	0.96
C4—H4B	0.97	C7—C8	1.354 (3)
C6—C7	1.373 (3)	C7—H7	0.93
C13—C12	1.492 (4)	C11—H11A	0.97
C13—H13A	0.97	C11—H11B	0.97
C13—H13B	0.97		
			
C5—N1—C1	124.27 (19)	H12A—C12—H12B	108.3
C5—N1—C4	123.00 (19)	C3—C2—C1	106.7 (2)
C1—N1—C4	112.31 (19)	C3—C2—H2A	110.4
C6—N2—C5	116.30 (18)	C1—C2—H2A	110.4
C6—N3—C13	124.4 (2)	C3—C2—H2B	110.4
C6—N3—C10	122.3 (2)	C1—C2—H2B	110.4
C13—N3—C10	113.3 (2)	H2A—C2—H2B	108.6
C8—N4—C5	115.7 (2)	N1—C1—C2	104.37 (19)
C2—C3—C4	106.1 (2)	N1—C1—H1A	110.9
C2—C3—H3A	110.5	C2—C1—H1A	110.9
C4—C3—H3A	110.5	N1—C1—H1B	110.9
C2—C3—H3B	110.5	C2—C1—H1B	110.9
C4—C3—H3B	110.5	H1A—C1—H1B	108.9
H3A—C3—H3B	108.7	N3—C10—C11	102.9 (2)
N1—C4—C3	103.21 (19)	N3—C10—H10A	111.2
N1—C4—H4A	111.1	C11—C10—H10A	111.2
C3—C4—H4A	111.1	N3—C10—H10B	111.2
N1—C4—H4B	111.1	C11—C10—H10B	111.2
C3—C4—H4B	111.1	H10A—C10—H10B	109.1
H4A—C4—H4B	109.1	C8—C9—H9A	109.5
N1—C5—N2	116.98 (19)	C8—C9—H9B	109.5
N1—C5—N4	118.4 (2)	H9A—C9—H9B	109.5
N2—C5—N4	124.6 (2)	C8—C9—H9C	109.5
N2—C6—N3	116.4 (2)	H9A—C9—H9C	109.5
N2—C6—C7	123.7 (2)	H9B—C9—H9C	109.5
N3—C6—C7	119.8 (2)	C8—C7—C6	116.7 (2)
N3—C13—C12	103.8 (2)	C8—C7—H7	121.7
N3—C13—H13A	111	C6—C7—H7	121.7
C12—C13—H13A	111	N4—C8—C7	123.0 (2)
N3—C13—H13B	111	N4—C8—C9	117.2 (2)
C12—C13—H13B	111	C7—C8—C9	119.8 (2)
H13A—C13—H13B	109	C12—C11—C10	110.0 (3)
C11—C12—C13	108.7 (3)	C12—C11—H11A	109.7
C11—C12—H12A	110	C10—C11—H11A	109.7
C13—C12—H12A	110	C12—C11—H11B	109.7
C11—C12—H12B	110	C10—C11—H11B	109.7
C13—C12—H12B	110	H11A—C11—H11B	108.2

### Hydrogen-bond geometry (Å, º)

**Table:**

*D*—H···*A*	*D*—H	H···*A*	*D*···*A*	*D*—H···*A*
C2—H2*B*···N3^i^	0.97 (1)	2.82	3.793 (2)	175
C9—H9*C*···N2^ii^	0.96 (1)	2.93	3.742 (2)	143

Symmetry codes: (i) −*x*, −*y*+1, −*z*; (ii) *x*−1, *y*, *z*.
